# Ni/(R_2_O_3_,CaO) Nanocomposites Produced by the Exsolution of R_1.5_Ca_0.5_NiO_4_ Nickelates (R = Nd, Sm, Eu): Rare Earth Effect on the Catalytic Performance in the Dry Reforming and Partial Oxidation of Methane

**DOI:** 10.3390/ma15207265

**Published:** 2022-10-18

**Authors:** Sergey A. Malyshev, Oleg A. Shlyakhtin, Alexey S. Loktev, Galina N. Mazo, Grigoriy M. Timofeev, Igor E. Mukhin, Roman D. Svetogorov, Ilya V. Roslyakov, Alexey G. Dedov

**Affiliations:** 1 Department of Chemistry, M.V. Lomonosov Moscow State University, 119991 Moscow, Russia; 2 Department of Materials Sciences, Shenzhen MSU-BIT University, Shenzhen 518172, China; 3 A.V. Topchiev Institute of Petrochemical Synthesis, Russian Academy of Sciences, 119991 Moscow, Russia; 4 Department of General and Inorganic Chemistry, Gubkin Russian State University of Oil and Gas, 119991 Moscow, Russia; 5 N.S. Kurnakov Institute of General and Inorganic Chemistry, Russian Academy of Sciences, 119991 Moscow, Russia; 6 National Research Center “Kurchatov Institute”, 123182 Moscow, Russia; 7 Department of Materials Sciences, M.V. Lomonosov Moscow State University, 119991 Moscow, Russia

**Keywords:** metal-oxide nanocomposites, multicomponent catalysts, rare earth effect, complex oxide precursors, K_2_NiF_4_ structure, exsolution, dry reforming of methane, partial oxidation of methane

## Abstract

In order to clarify the role of R_2_O_3_ in the metal-oxide catalysts derived from complex oxide precursors, a series of R_1.5_Ca_0.5_NiO_4_ (R = Nd, Sm, Eu) complex oxides was obtained. A significant systematic increase in the orthorhombic distortion of the R_1.5_Ca_0.5_NiO_4_ structure (K_2_NiF_4_ type, Cmce) from Nd to Eu correlates with a corresponding decrease in their ionic radii. A reduction of R_1.5_Ca_0.5_NiO_4_ in the Ar/H_2_ gas mixture at 800 °C causes a formation of dense agglomerates of CaO and R_2_O_3_ coated with spherical 25–30 nm particles of Ni metal. The size of metal particles and oxide agglomerates is similar in all Ni/(R_2_O_3_,CaO) composites in the study. Their morphology is rather similar to the products of redox exsolution obtained by the partial reduction of complex oxides. All obtained composites demonstrated a significant catalytic activity in the dry reforming (DRM) and partial oxidation (POM) of methane at 700–800 °C. A systematic decrease in the DRM catalytic activity of composites from Nd to Eu could be attributed to the basicity reduction of R_2_O_3_ components of the composite catalysts. The maximum CH_4_ conversion in POM reaction was observed for Ni/(Sm_2_O_3_,CaO), while the maximum selectivity was demonstrated by Nd_2_O_3_-based composite. The possible reasons for the observed difference are discussed.

## 1. Introduction

Most of the modern catalysts of methane conversion to synthesis gas consist of the nickel nanoparticles allocated at the surface of the various oxide substrates by means of the traditional incipient wetness technique [1,2,3,4]. The application of this technique to the synthesis of the modern multicomponent metal-oxide catalysts is rather complicated. In order to solve this problem, several alternative chemical synthesis methods are currently in study. They are based on the reductive decomposition of various precursors containing all the necessary cations of these composites in a single molecule or a chemical compound [5,6,7,8]. These techniques ensure the homogeneous spatial distribution and the tight contact of the particles of individual components in the decomposition products.

One of the most promising of these alternative methods is a reduction of complex oxides by hydrogen at elevated temperatures. In the case of the partial reduction of complex oxides, also called a redox exsolution technique, the reduction products consist of the spherical nanoparticles of metal exsolved from the volume of oxide precursor and tightly bound to the surface of micron-sized particles of the partially reduced complex oxides [9,10,11,12,13,14]. The main advantages of this synthesis technique deal with the enhanced adhesion of metal nanoparticles to the oxide substrate and the possibility of obtaining multicomponent substrates often used in modern catalytic systems. It was recently discovered that similar nanocomposites could be obtained not only from the partial but also from the complete reduction of complex oxides. In this case, reductive decomposition of the complex oxide causes the formation of tightly bound crystallites of the individual oxides covered by the spherical metal nanoparticles [15,16]. Both kinds of reduction products appear to be suitable for application in redox catalysis at elevated temperatures.

The most widely used group of these precursors is complex oxides with a perovskite structure due to their relatively simple synthesis at moderate temperatures and high stability of these compounds at elevated temperatures for exsolution synthesis [17,18,19]. However, in spite of a wide variety of perovskites, the amount of Ni-containing complex oxides with this structure is rather limited. This constraint deals with the limited stability of the perovskite lattice, usually described as a Goldschmidt’s tolerance rule strictly limiting the allowed oxidation states and ionic radii of A cations in the ANiO_3_ lattice. For these reasons, most studies on the perovskite-derived Ni-based catalysts deal with LaNiO_3_ and its solid solutions as precursors. The possibility of obtaining nanocomposites of Ni metal with other oxides from these precursors does not seem reasonable.

These limitations produce serious complications to finding the optimum oxide components of the metal-oxide catalyst. It is known that the oxide substrate or, more generally, the oxide components of the composite Ni/MeO_x_ catalysts have a significant effect on their catalytic performance due to both their own acid–base and redox properties and due to the metal–substrate interactions (MSI) [20,21]. In the case of methane conversion, the application of oxides with an acidic surface in catalysis is not desirable, as it prevents the sorption of CO_2_ in the course of the complex reaction of methane conversion at the surface of catalyst; it also promotes the intense coke formation during its exploitation. For these reasons it is recommended to use metal oxides with significant or strong basicity, like rare earth and/or alkaline earth oxides, in these catalysts [1,22,23,24,25,26]. In order to promote the redox processes at the surface of catalyst, the application of oxides with significant oxygen mobility in their lattice is also helpful. However, the optimum selection of these oxides and their combinations to improve the performance of conversion catalysts is still under consideration.

The application of Ruddlesden–Popper complex oxides as precursors for the Ni/MeO_x_ catalysts opens new ways to study the effect of various rare earth and alkaline earth oxides on the physico-chemical properties and catalytic performance of these composites [27,28,29]. These (R,A)_2_NiO_4_ compounds are known for the several light rare earth elements such as Gd. Due to the stability criteria of K_2_NiF_4_-like phases, the partial substitution of R with Ca in these nickelates promotes the stabilization of these compounds and allows one to obtain them at lower temperatures [30,31,32,33,34,35]. The formation of continuous R_2−x_Ca_x_NiO_4_ solid solutions allows one to obtain Ni/(R_2_O_3_,CaO) composites with various R/Ca ratios from single-phase precursors under the same processing conditions, ensuring correct comparison of their physico-chemical and catalytic properties.

It is shown during these studies that the maximum catalytic activity in the POM reaction among Ni/(Nd_2_O_3_,CaO) composites is demonstrated by the Ca-free Ni/Nd_2_O_3_ counterpart. However, its activity in the DRM reaction is found to be less than that of the others. Meanwhile, the optimum selection of a rare earth element for application in these catalysts with specific morphology remains unclear. For these reasons a synthesis of several R_2−x_Ca_x_NiO_4_ precursors (R = Nd, Sm, Eu) is performed in order to compare the morphology and the catalytic properties of their reduction products in partial oxidation and dry reforming of methane.

## 2. Materials and Methods

### 2.1. Synthesis

In order to obtain R_1.5_Ca_0.5_NiO_4_ (R = Nd, Sm, Eu) by the freeze-drying synthesis method Nd_2_O_3_, Sm_2_O_3_, Eu_2_O_3_, CaCO_3_, and Ni(NO_3_)_2_·6H_2_O were used as precursors. Nd_2_O_3_, Sm_2_O_3_, and Eu_2_O_3_ were annealed at 800 °C for 2 h and CaCO_3_ at 400 °C for 1 h before use in order to remove the adsorbed H_2_O. The amount of H_2_O in nickel nitrate was refined by the gravimetric analysis. R_2_O_3_ and CaCO_3_ in the stoichiometric ratios were dissolved in 20% acetic acid; corresponding amount of Ni(NO_3_)_2_·6H_2_O was added. An aqueous solution (5 mass %) of polyvinyl alcohol was added to all solutions under intense stirring. The freeze-drying of flash-frozen solutions was performed in a Labconco FreeZone 7948030 tray dryer (Labconco, Kansas City, MI, USA) at P = 0.7 mbar for 2 days. Thermal decomposition of the freeze-dried products was performed in air at 1200 °C for 6 h. A reduction of as-obtained R_1.5_Ca_0.5_NiO_4_ powders was performed in an H_2_:Ar = 1:20 gas mixture at 850 °C for 1 h followed by slow cooling to room temperature.

### 2.2. Characterization

XRD analysis of the powders was performed using a Rigaku D/MAX-2500PC diffractometer (Rigaku, Tokyo, Japan) with Cu K_α1_ radiation generated on a rotating Cu anode (40 kV, 250 mA). More detailed investigation of the R_1.5_Ca_0.5_NiO_4_ crystal structure was performed using powder diffraction of synchrotron radiation at a wavelength λ = 0.74 Å. The measurements were performed using a 2D Rayonix SX165 detector (Rayonix LLC, Evanston, IL, USA) at the XSA (X-ray Structural Analysis) beamline of the Kurchatov synchrotron radiation source. The Rietveld crystal structure refinement of the XRD data was carried out by the Jana 2006 program package.

The temperature-program med reduction (H_2_-TPR) of R_1.5_Ca_0.5_NiO_4_ oxides was performed using a USGA device in an H_2_:Ar = 1:20 gas mixture at a flow rate of 30 cm^3^ min^−1^. The temperature of the samples (~0.05 g) was increased to 950 °C at a heating rate of 5 °C min^−1^. The morphology of the powders was studied using a Carl Zeiss NVision 40 scanning electron microscope (Carl Zeiss SMT AG, Oberkochen, Germany).

### 2.3. Catalytic Experiments

The catalytic tests of the DRM and POM reactions were carried out in a quartz glass flow fixed-bed reactor (18 mm internal diameter, 300 mm length). The temperature inside the reactor was measured by a thermocouple placed in a special pocket running lengthwise along the reactor axis, 8 mm in diameter. A 0.2 g sample of catalyst (100–250 mesh fraction) was placed in the middle part of the reactor between two quartz glass rods. The free space in the reactor was filled with closely packed quartz glass fillers in order to eliminate gas-phase reactions outside the catalyst. The catalytic tests were carried out at atmospheric pressure in the absence of dilution with inert gas. The catalyst was first heated in hydrogen flow at 10 °C min^−1^ to 900 °C. Then, the gas stream was switched to a mixture of CH_4_/CO_2_ = 1/1 or CH_4_/O_2_ = 2/1. According to the results of our previous studies (Figure 3 in [36]), the GHSV values were set at 16 and 12 L g^−1^ h^−1^ for DRM and POM, respectively. No dilution of the feed flow by the inert gas was applied. The catalytic experiments were performed consecutively at 900, 800, 700, and 600 °C by maintaining the preselected temperatures for 1–5 h. After the analysis, the furnace was switched off, and the catalyst was cooled to room temperature over 3–4 h in pure N_2_.

The methane conversion (*X*), product selectivity (*S*), and yield (*Y*) of the products are defined as follows:

(1) $$X\left(CH_{4},\%\right)=\frac{moles\cdot of\cdot CH_{4}\cdot converted}{moles\cdot of\cdot CH_{4}\cdot in\cdot feed}\times 100$$

(2) $$S\left(CO\cdot or\cdot CO_{2},\%,\cdot POM\right)=\frac{moles\cdot of\cdot CO\cdot in\cdot products}{moles\cdot of\cdot CH_{4}\cdot converted}\times 100$$

(3) $$\begin{array}{l} S\left(CO,\%,\cdot DRM\right)=\frac{moles\cdot of\cdot CO\cdot in\cdot products}{moles\cdot of\cdot CH_{4}+CO_{2}\cdot converted}\times 100 \end{array}$$

(4) $$S\left(H_{2},\%\right)=\frac{moles\cdot of\cdot H_{2}\cdot produced}{2\times moles\cdot of\cdot CH_{4}\cdot converted}\times 100$$

(5) $$Y\left(products,\%\right)=\frac{X\left(CH_{4},\%\right)\cdot \times \cdot S\left(products,\%\right)}{100}$$

(6) $$C\;balance\;\left(\%\right)=\frac{moles\cdot of\cdot C\cdot in\;products}{moles\cdot of\cdot C\cdot in\cdot feed}\times 100$$

The number of moles of the feed gases and gaseous products of the reactions was calculated based on the measured volumetric velocity of the feeder gases and the products formed, as well as chromatography data, which makes it possible to fully take into account the stoichiometry of the reaction and the corresponding volume of expansion of the gaseous mixture of reagents.

## 3. Results and Discussion

### 3.1. Synthesis of R_1.5_Ca_0.5_NiO_4_

**Figure 1:**
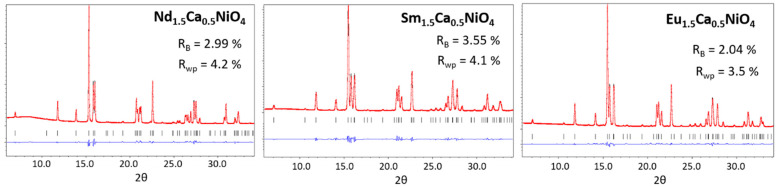
Rietveld refinement plots of the synchrotron data for Nd_1.5_Ca_0.5_NiO_4_, Sm_1.5_Ca_0.5_NiO_4_, and Eu_1.5_Ca_0.5_NiO_4_: observed (red), calculated (black) and difference (blue) curves. Bragg reflections positions are marked as short vertical lines below the observed and calculated data.

Single-phase Nd_1.5_Ca_0.5_NiO_4_, Sm_1.5_Ca_0.5_NiO_4_, and Eu_1.5_Ca_0.5_NiO_4_ nickelates were obtained using a freeze-drying procedure similar to that in [15,16]. XRD study of the obtained complex oxides revealed that their crystal structure belonged to the orthorhombically distorted K_2_NiF_4_ type. Detailed investigation of the obtained R_1.5_Ca_0.5_NiO_4_ crystal structure was performed using Rietveld refinement of the synchrotron powder diffraction data (Figure 1).

The orthorhombic Cmce model was assigned to each nickelate. This polymorph of the K_2_NiF_4_ structure was previously observed for Nd_2−x_Ca_x_NiO_4_ solid solutions with Ca contents close to 0.5 [16]. In addition, the same structure type was found for Sm_1.5_Ca_0.5_NiO_4_ oxide in [37]. Information on the structure and properties of Eu_1.5_Ca_0.5_NiO_4_ was not found in the literature.

**Figure 2:**
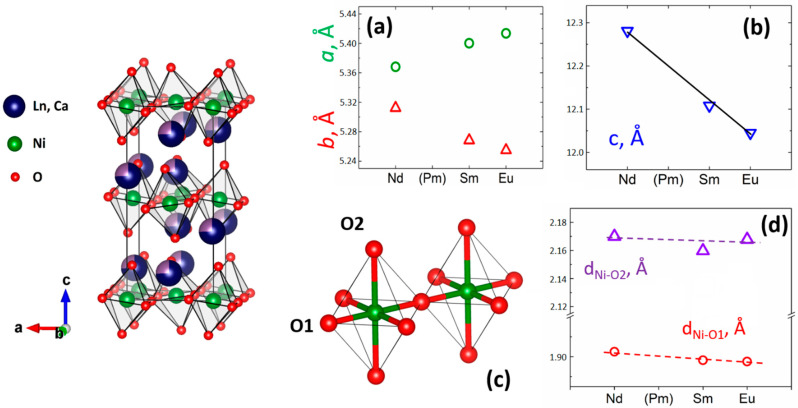
Representation of the Cmce lattice of the R_1.5_Ca_0.5_NiO_4_ crystal structure obtained by the Rietveld refinement (left); dependences of lattice parameters on the nickelate composition (**a**,**b**); representation of Ni octahedral coordination in the R_1.5_Ca_0.5_NiO_4_ structure obtained by the Rietveld refinement (**c**); dependences of Ni-O distances on the nickelate composition (**d**).

According to the Rietveld refinement data, the R_1.5_Ca_0.5_NiO_4_ unit cell *c* parameter decreased systematically from Nd to Eu (Figure 2a,b) in accordance with the decrease of the R^3+^ ionic radii [31,38]. The degree of orthorhombic distortion which can be estimated by the difference between the *a* and *b* parameters was found to increase in this series (Figure 2a). However, despite such considerable changes in unit cell dimensions, the observed Ni-O distances were almost the same within the R_1.5_Ca_0.5_NiO_4_ series (Figure 2c,d). This indicated that the valence states of Ni were highly likely to be the same for all discussed R_1.5_Ca_0.5_NiO_4_ compounds and similar to that for Nd_1.5_Ca_0.5_NiO_4_ wherein the mixed Ni^2+^:Ni^3 +^ =1:1 valence state of Ni was proposed [15]. Moreover, the fact that all of the R_1.5_Ca_0.5_NiO_4_ nickelates were characterized by the Cmce type of K_2_NiF_4_ structure might be attributed to the nearly stoichiometric oxygen content in these complex oxides also observed for Nd_1.5_Ca_0.5_NiO_4_, and thus correspond to the Ni formal oxidation state of 2.5 [39].

Therefore, the structure distortion in Nd-Sm-Eu nickelates is likely related to the corresponding rare earths’ ionic radii effect, similar to that in ABO_3_ perovskites described by Goldschmidt’s tolerance factor. Smaller R^3+^ cations corresponded to less-stable perovskite-like K_2_NiF_4_ oxides. This decrease in the K_2_NiF_4-_like lattice stability correlates with a systematic increase in the temperature needed to obtain Nd_1.5_Ca_0.5_NiO_4_, Sm_1.5_Ca_0.5_NiO_4_, and Eu_1.5_Ca_0.5_NiO_4_ nickelates (1000, 1100, and 1250 °C, respectively). Gd_1.5_Ca_0.5_NiO_4_ is less stable, so we could not obtain it even at 1350 °C (Supplementary Figures S1 and S2).

### 3.2. Synthesis of Ni/(R_2_O_3_,CaO) Composites

**Figure 3:**
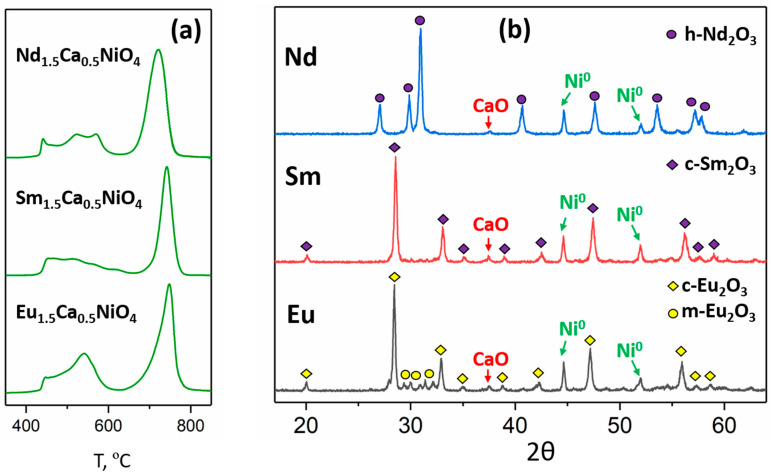
H_2_-TPR profiles for Nd_1.5_Ca_0.5_NiO_4_, Sm_1.5_Ca_0.5_NiO_4_, and Eu_1.5_Ca_0.5_NiO_4_ (**a**); XRD plots of the nickelates’ reduction products at 900 °C (**b**).

According to the temperature-programmed reduction (H_2_-TPR) data, the reduction of the Sm_1.5_Ca_0.5_NiO_4_ and Eu_1.5_Ca_0.5_NiO_4_ complex oxides occurred in a similar way to that of Nd_1.5_Ca_0.5_NiO_4_; the latter was described in [15]. All three H_2_-TPR profiles consisted of two maxima of H_2_ consumption, one at 450–600 °C and one at 700–800 °C (Figure 3a). These maxima can be attributed to the partial and complete reduction of the complex oxide, respectively. According to previous research data [15,16], the complete reduction of K_2_NiF_4_ nickelates led to the mixture of Ni metal and individual rare/alkaline earths’ oxides; this was the case for Sm_1.5_Ca_0.5_NiO_4_ and Eu_1.5_Ca_0.5_NiO_4_, too. Analysis of the XRD data showed that all of the nickelates under investigation were completely reduced at 900 °C in H_2_ flow. All of the reduced samples were composed of Ni metal (ICCD#: 00-004-0850), CaO (ICCD#: 00-037-1497), and different polymorphs of R_2_O_3_ (Figure 3b). For the Nd-containing sample, it was h-Nd_2_O_3_ (ICCD#: 00-041-1089); for the Sm-containing sample, it was c-Sm_2_O_3_ (ICCD#: 00-015-0813); and for Eu-containing sample, it was a mixture of cubic (ICCD#: 00-034-0392) and monoclinic (ICCD#: 00-034-0072) Eu_2_O_3_ modifications.

The microstructure of the obtained Ni/(R_2_O_3_,CaO) composites was investigated using the SEM technique. It has been established that the morphology transformations during the R_1.5_Ca_0.5_NiO_4_ complete reduction were similar to those observed in the case of Nd_2−x_Ca_x_NiO_4_ and Nd_2−y_Ca_y_Co_1−x_Ni_x_O_4_ reduction [15,16,36].

**Figure 4:**
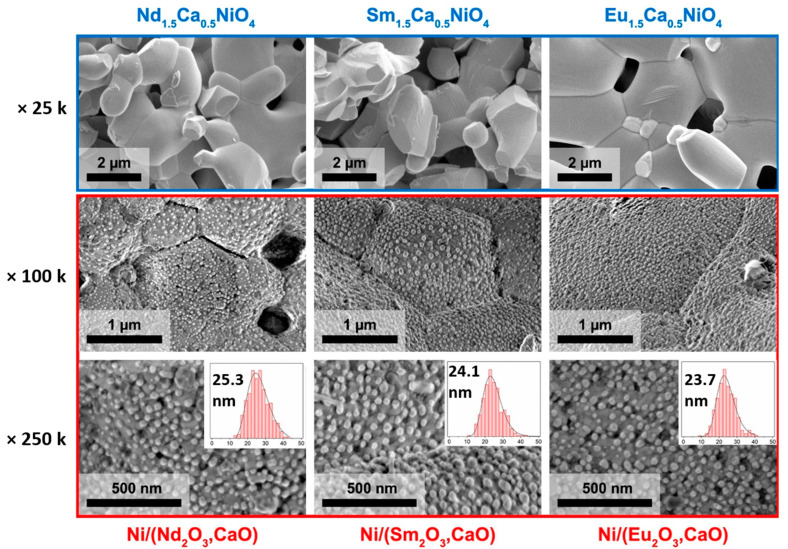
SEM micrographs of the initial R_1.5_Ca_0.5_NiO_4_ oxides (×25k) and nanocomposites obtained by the reduction of the corresponding nickelates at 900 °C (×100k; ×250k).

According to the SEM micrographs, the initial R_1.5_Ca_0.5_NiO_4_ samples consisted of 2–3 μm crystallites with clear traces of the intense sintering which was induced by the relatively high annealing temperatures that occurred during their synthesis (Figure 4, ×25k). The composites obtained by the reduction of R_1.5_Ca_0.5_NiO_4_ were also characterized by a similar ceramic-like morphology. They were constructed by large, closely packed 2–3 μm grains of nearly polygonal shape separated by the distinct grain boundaries; the surface of such grains was uniformly covered by spherical nanoparticles.

This type of morphology is usually observed for redox exsolution products, wherein the grains of the partially reduced oxide precursors are decorated with uniformly distributed metal nanoparticles [11,12,13,14]. The same microstructural pattern has been observed for complete reduction products of Nd_2−x_Ca_x_NiO_4_, for which ~25 nm Ni particles were anchored to the surface of dense agglomerates of Nd_2_O_3_ and CaO oxides [16]. These composites inherited the morphology of the initial nickelate powder. In the present study, Ni/(Nd_2_O_3_,CaO) composite also inherited the morphology of the sintered Nd_1.5_Ca_0.5_NiO_4_ sample. According to our previous studies [15,16], these large “grains” corresponded to the dense aggregates of Nd_2_O_3_ and CaO, while spherical particles anchored to their surface corresponded to the Ni metal phase. The same microstructure was observed for the Ni/(Sm_2_O_3_,CaO) and Ni/(Eu_2_O_3_,CaO) composites obtained by the reduction of Sm_1.5_Ca_0.5_NiO_4_ and Eu_1.5_Ca_0.5_NiO_4_, respectively, which has never been described before.

Statistical analysis of the Ni particles size in SEM micrographs of the Ni/(R_2_O_3_,CaO) composites (Figure 4; ×100k, ×250k) demonstrated that all of the samples were characterized by similar Ni size distributions, with maxima around 24 nm and little or no rare earth effect. This shows the primary role of the similar R_1.5_Ca_0.5_NiO_4_ reduction conditions detected by the TPR technique and the equal H_2_ annealing temperatures that led to the similar morphology of the Ni/(R_2_O_3_,CaO) composites.

### 3.3. DRM and POM Catalytic Testing

**Figure 5:**
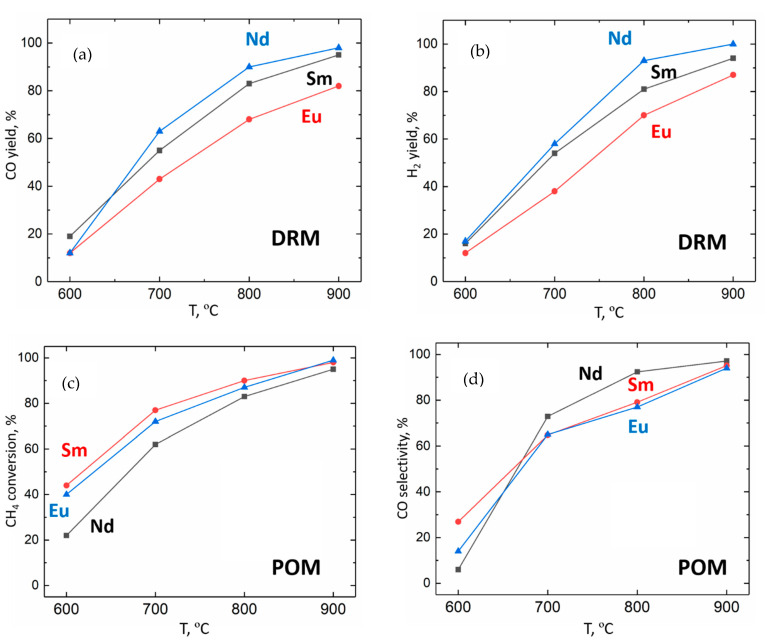
The dependences of CO and H_2_ yields in the DRM reaction on the temperature for Ni/(R_2_O_3_,CaO) nanocomposites (**a**,**b**). Dependences of CH_4_ conversion and CO selectivity in the POM reaction on the temperature for Ni/(R_2_O_3_,CaO) nanocomposites (**c**,**d**).

The catalytic performance of the obtained Ni/(R_2_O_3_,CaO) nanocomposites in DRM and POM processes at 600–900 °C was evaluated using a flow reactor and undiluted CH_4_/CO_2_ and CH_4_/O_2_ mixtures, respectively. It was found during DRM testing that all of the presented samples demonstrated relatively high catalytic activity compared with other Ni-based catalysts (Figure 5a,b) [1,2]. However, the values of the CO and H_2_ yields decreased within the Nd-Sm-Eu series from ~90 % for the Nd-containing sample to ~70% for the Eu-containing catalyst at 800 °C.

The opposite tendency was observed during POM testing of the Ni/(R_2_O_3_,CaO) nanocomposite catalysts (Figure 5c,d). Ni/(Sm_2_O_3_,CaO) and Ni/(Eu_2_O_3_,CaO) samples demonstrated slightly higher CH_4_ conversion than the Nd-containing catalyst. Notably, that Ni/(Nd_2_O_3_,CaO) nanocomposite showed better CO selectivity at 700 and 800 °C in the POM reaction.

**Figure 6:**
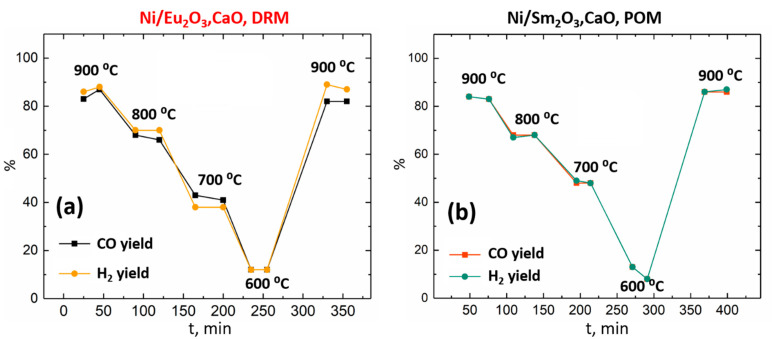
Temporal dependence of CO and H_2_ yields in DRM (**a**) and POM (**b**) reactions at different temperatures for Ni/(Eu_2_O_3_,CaO) and Ni/(Sm_2_O_3_,CaO) catalysts, respectively.

The essential feature of such metal–oxide composites produced by the exsolution-like synthesis is the reproducibility of their catalytic performance in the course of redox processes taking place in the active phase. It was determined that this was the case for R_1.5_Ca_0.5_NiO_4_ reduction products. It was reported previously [16] that Ni/(Nd_2_O_3_,CaO) nanocomposites obtained via Nd_2−x_Ca_x_NiO_4_ decomposition remained chemically stable during DRM reaction at 600–900 °C, whereas the POM environment caused the oxidation of Ni particles to NiO oxide. Thus, the same tendency is likely presented in the case of Ni/(R_2_O_3_,CaO) catalysts. Cyclic DRM catalytic testing (Figure 6a) revealed that the high performance of Ni/(Eu_2_O_3_,CaO) nanocomposite was completely restored when the reaction temperature was raised back to 900 °C. Cyclic POM testing of Ni/(Sm_2_O_3_,CaO) catalyst (Figure 6b) also demonstrated a similar restoration of the CO and H_2_ yields to their high initial values after being reheated, despite the oxidation processes that took place at lower temperatures. It is notable that the decrease of R_1.5_Ca_0.5_NiO_4_ stability discussed before in Section 3.1 should completely suppress the resynthesis process, which drastically decreased the catalytic activity of the similar Co-containing composites in the POM reaction [15].

Since the microstructures of all of the obtained materials were found to be similar, the difference in the catalytic DRM and POM performance can likely be attributed to the difference in their Ni/(R_2_O_3_,CaO) compositions. The possible effects of various rare earth oxides on the catalytic activity in DRM and POM reactions were discussed in [23,24,25]. In most cases, the role of R_2_O_3_ oxide in DRM reactions is usually associated with CO_2_ capture and activation. Thus, a higher basicity of the rare earth oxide provides more efficient CO_2_ chemisorption, leading to higher catalytic activity. As the basicity decreases in the Nd_2_O_3_–Sm_2_O_3_–Eu_2_O_3_ series, it seems reasonable to suppose a parallel decrease in the CO_2_ activation efficiency. This is likely the case for exsolved Ni/(R_2_O_3_,CaO) nanocomposites-containing samples—both CO and H_2_ yields decrease from Nd to Eu.

In the case of POM reactions, the roles of the rare earth oxides are different at the different stages of this complex multistage process. These oxides are often considered as a source of active lattice oxygen, providing total oxidation of methane to CO_2_—the first step to the partial oxidation products. For instance, the role of La_2_O_3_ in POM reactions over Ni/La_2_O_3_ catalyst is discussed in detail in [28]; the involvement of Nd_2_O_3_ lattice oxygen is also reported in [40]. Thus, the observed differences in the CH_4_ conversion between Ni/(Nd_2_O_3_,CaO) and Sm- and Eu-containing catalysts can be attributed to the different efficiencies of total oxidation. Most likely, Sm_2_O_3_ and Eu_2_O_3_ provide a higher yield of the intermediate CH_4_ → CO_2_ transformation in comparison with Nd_2_O_3_ oxide. This effect led to the slight but distinct increase in the methane conversion values at 700–800 °C. On the other hand, the increase in CO_2_ yield in the first POM reaction step can cause an increase in the overall CO_2_ yield, lowering the CO selectivity. This hypothesis was found to be in good agreement with the experimental data; CO selectivity values for Ni/(Nd_2_O_3_,CaO) at 800 °C are ~20 % higher than those for Ni/(Sm_2_O_3_,CaO) and Ni/(Eu_2_O_3_,CaO) nanocomposites.

## 4. Conclusions

Nd_1.5_Ca_0.5_NiO_4,_ Sm_1.5_Ca_0.5_NiO_4_, and Eu_1.5_Ca_0.5_NiO_4_ nickelates with perovskite-like K_2_NiF_4_ structure were synthesized by freeze-drying technique; Eu_1.5_Ca_0.5_NiO_4_ was obtained and described for the first time. Rietveld refinement of the synchrotron diffraction data proved the formation of Cmce structure modification with a gradual increase of the orthorhombic distortion grade from Nd- to Eu-containing oxide.

All of the obtained nickelates demonstrated similar complete reduction conditions determined using the H_2_-TPR technique. The reduction processes of Sm_1.5_Ca_0.5_NiO_4_ and Eu_1.5_Ca_0.5_NiO_4_ and the properties of their reduction products are also described for the first time. The composites obtained by R_1.5_Ca_0.5_NiO_4_ reduction at 900 °C consisted of Ni metal, CaO, and corresponding R_2_O_3_ oxides. The microstructure of as-obtained metal-oxide nanocomposites is very similar to the typical microstructure of the redox exsolution products: the crystallites of the oxide phases formed dense agglomerates decorated by the uniformly distributed ~25 nm anchored particles of Ni metal. The identical microstructure of all of the Ni/(R_2_O_3_,CaO) nanocomposites in the study can be attributed to the similar temperatures of their reduction.

Catalytic testing of the obtained Ni/(R_2_O_3_,CaO) materials in DRM and POM reactions proved their excellent activity in both processes. Comparative analysis of their catalytic properties demonstrated a gradual decrease of the syngas yield in the DRM process within the Ni/(Nd_2_O_3_,CaO)–Ni/(Sm_2_O_3_,CaO)–Ni/(Eu_2_O_3_,CaO) series. This effect is most likely related to the decrease of the corresponding rare earth oxides’ basicity and less-efficient CO_2_ activation. During POM testing, a slight increase in the methane conversion was detected for the same series. The probable nature of this effect could deal with the intensity increase of the total oxidation of methane, which led to the considerable decrease in the CO selectivity of Ni/(Sm_2_O_3_,CaO) and Ni/(Eu_2_O_3_,CaO) in comparison with the Ni/(Nd_2_O_3_,CaO) sample.

## Supplementary Materials

The following supporting information can be downloaded at: https://www.mdpi.com/article/10.3390/ma15207265/s1.
