# Supplementary material for: Ni/(R2O3,CaO) Nanocomposites Produced by the Exsolution of R1.5Ca0.5NiO4 Nickelates (R = Nd, Sm, Eu): Rare Earth Effect on the Catalytic Performance in the Dry Reforming and Partial Oxidation of Methane
Source: Materials (Basel). 2022 Oct 18;15(20):7265. doi: 10.3390/ma15207265 (PMC9610205; doi:10.3390/ma15207265)
Supplement: Supplementary file 1 [file materials-15-07265-s001.zip › materials-1914594-supplementary.pdf]

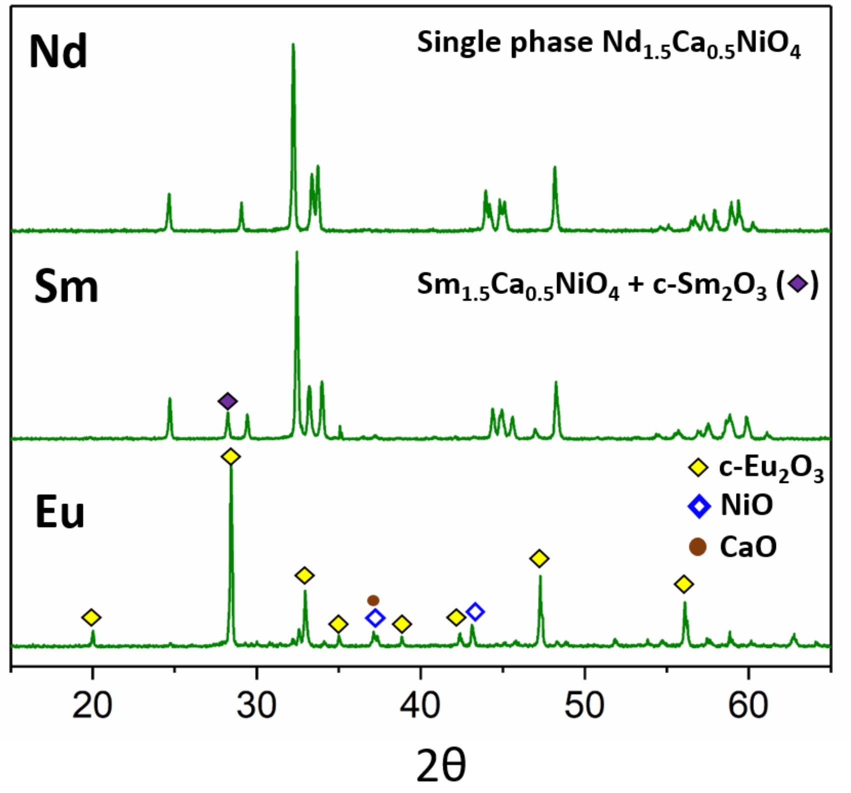

Figure S1. XRD plots of $R_{1.5}Ca_{0.5}NiO_4$ samples obtained at 1000 ^{o}C. Crystalline phases have been assigned based on following ICCD data: c-Sm_2O_3 (# 00-015-0813), c-Eu_2O_3 (# 00-034-0392), NiO (# 00-047-1049) and CaO (# 00-037-1497)

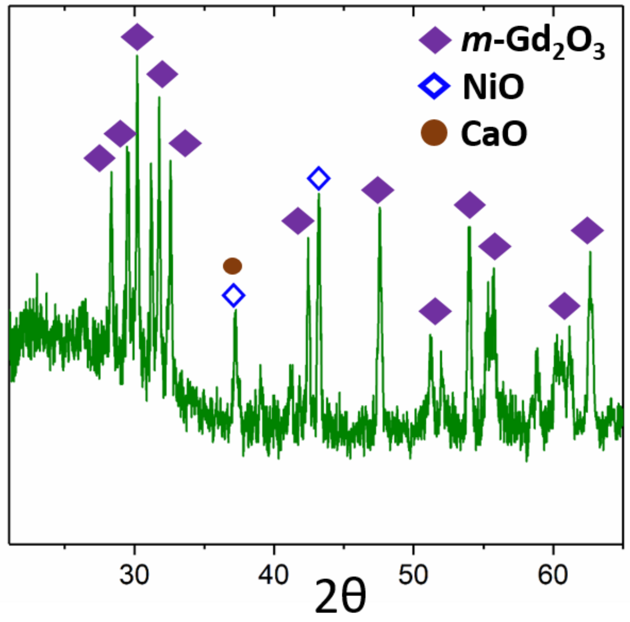

Figure S2. XRD plot of "$Gd_{1.5}Ca_{0.5}NiO_4$" sample obtained at 1350 ^{o}C. Crystalline phases have been assigned based on following ICCD data: m-Gd_2O_3 (# 00-042-1465), NiO (# 00-047-1049) and CaO (# 00-037-1497)
